# A narrative review of present knowledge and digital approaches in orthognathic surgery

**DOI:** 10.1515/iss-2024-0018

**Published:** 2024-09-06

**Authors:** Constanze Friedrich, Constantin Graw, Juliane Kröplin

**Affiliations:** Department of Oral and Maxillofacial Surgery, University Medical Centre Rostock, Rostock, Germany

**Keywords:** surgeries, approaches, orthognathic, surgical, planning, treatment

## Abstract

**Introduction:**

Anomalies of jaw position and shape affect approximately 10 % of the population. They can have a significant impact on quality of life, which is why the continuous improvement of therapeutic approaches is a key concern in oral and maxillofacial surgery. The aim of this narrative review article is to examine the development of orthognathic surgery in the context of traditional and innovative methods.

**Content:**

A Pubmed-based selective literature search was performed considering literature predominantly from 2022 to 2023. Search terms were “orthognathic surgery” in combination with “virtual surgical planning” and “3D printing”.

**Summary:**

Depending on the extent of the existing anomalies, there are purely orthodontic or combined orthodontic-surgical treatment approaches. Technological innovations in particular are changing both the therapeutic approach and the planning of surgical treatment to an almost completely digital workflow. This change can lead to greater precision in treatment and more efficient planning, resulting in reduced costs and an overall improvement in clinical outcomes, including patient satisfaction.

**Outlook:**

This study presents an overview of the field of orthognathic surgery and discusses developments and challenges for the future. With traditional approaches being time-consuming and prone to error digital technologies like VSP, AI and PSIs improve accuracy and efficiency, though challenges persist.

## Introduction - etiology and need for treatment

Orthognathic surgery is a branch of oral and maxillofacial surgery. Various surgical techniques can be used to correct anomalies in the shape and position of the jaws. The first description of a mandibular osteotomy to correct mandibular prognathism, a malposition with an elongated mandible, dates back to 1849 by Simon P. Hullien. Due to new surgical techniques, technical achievements and changing patient needs, this field has evolved enormously since then and has been undergoing constant change, especially in recent decades. Anomalies in the shape and position of the jaws can be acquired or inherited. Their frequency is estimated at around 10 % of the population, although exact figures are not available [1]. They can significantly impair various functions such as breathing, swallowing, speaking and chewing. Oral health and psychosocial well-being can also be negatively affected as a result of an inharmonious appearance. The causes of skeletal dysgnathia are variable. In childhood, premature loss of milk teeth, sucking habits or mouth breathing can lead to a misdirected growth stimulus with corresponding developmental disorders of the facial skull. In addition to a proven hereditary component in true progenia, hormonal influences and fractures in the facial region can be the cause of a disharmonious growth pattern of the upper and lower jaw. Another group of patients who can benefit from orthognathic surgery are patients with syndromic diseases. These include syndromes associated with cleft lip and palate, hemifacial microsomia and Franceschetti syndrome 2], [3], [4], [5.

## Methods

A focused literature search was conducted using PubMed, emphasizing publications predominantly from 2022 to 2023. The search targeted mainly open access articles discussing current trends and new evidence-based strategies, using the specific search terms “orthognathic surgery” combined with “virtual surgical planning” and “3D printing.” Given the selective nature of the search, strict criteria were not applied, allowing for flexibility in literature collection. The included literature comprised scoping reviews, prospective cohort studies, narrative reviews, teaching books, retrospective studies, and systematic reviews. A total of 17 studies have been included overall. While the primary focus was on recent advancements and innovations, a few older articles were included to provide foundational information and enhance the comprehensibility of the specialty for a broader audience.

## Therapeutic treatments

Especially in adolescent patients with a rather low degree of jaw disproportion, orthodontic treatment is the treatment of choice. Fixed or removable orthodontic appliances are used to achieve dental compensation or to compensate for the disproportion of the jaws by utilizing and controlling growth. If orthodontic procedures alone are not sufficient to achieve a satisfactory occlusion and jaw relationship, surgical treatment is also required. Through early interdisciplinary coordination of orthodontic and oral surgery treatment techniques, it is now possible to achieve a harmonious external appearance with a balanced and stable occlusion. Depending on the skeletal anomaly, either one or both jaws are repositioned as part of the surgical treatment and then fixed in position, usually using mini plates. This can involve a forward or backward shift, a horizontal or vertical correction and asymmetries can also be corrected. Finally yet importantly, orthognathic surgery can be used not only to correct skeletal anomalies, but also to harmonize soft tissue (e.g. with gummy smile) [2, 4].

## Traditional approach

Traditional orthognathic surgery involves a preoperative and postoperative orthodontic treatment in addition to the surgical phase (“sandwich technique”). On average, the total treatment time is therefore up to 36 months between the start of preoperative and the end of postoperative orthodontic treatment. In traditional surgical planning (TSP), multiple data, including two-dimensional X-rays (lateral cephalogram, orthopantogram) and facial photographs, are required to plan the surgical treatment. Dental impressions and a facebow are used to produce dental stone models that represent the current relationship between the upper and lower jaw in an articulator. A model operation is performed on these models to evaluate the planned movements of the upper and lower jaw and to produce surgical guides. These guides are then used during the operation to transfer the movements planned in the model to the patient intraoperatively. During this process, close cooperation with a dental laboratory with appropriately trained staff is necessary. Depending on the distance between the clinic and laboratory, the transportation of the models alone may result in waiting times that can delay treatment. This approach is time-consuming, depends on specialist expertise at various points and is also prone to errors [3, 6, 7].

## LeFort I osteotomy

The LeFort I osteotomy is a widely accepted surgical technique used to correct midfacial deformities. This approach involves a horizontal osteotomy above the teeth, allowing for the repositioning of the maxilla. It is commonly used to address issues such as maxillary hypoplasia, open bite, and other midface discrepancies. The procedure enables three-dimensional movement of the maxilla, which can be advanced, set back, impacted, or down grafted as needed to achieve the desired functional outcomes and facial aesthetics [1].

## Retromolar approach (Obwegeser–Dalpont)

The retromolar approach, as described by Obwegeser and later modified by Dalpont, is primarily used for mandibular ramus osteotomies. This technique involves making an incision in the retromolar region, allowing for access to the mandibular ramus and facilitating sagittal split osteotomies. The retromolar approach is particularly advantageous for its ability to provide excellent visualization and access to the surgical site, minimize nerve injury risk, and offer stable postoperative results. It is commonly employed in the correction of mandibular prognathism and retrognathism, as well as other mandibular asymmetries [1].

## Differentiation between maxilla-first and mandible-first approaches

The decision to perform a maxilla first or mandible first approach depends on various clinical factors, including the specific deformity being corrected and the surgeon’s preference.

The maxilla-first approach involves repositioning the maxilla before addressing the mandible. This sequence is often preferred in cases where the maxillary position is crucial for achieving optimal occlusion and facial aesthetics. By first establishing the correct position of the maxilla, surgeons can more accurately plan and execute the mandibular movements needed to achieve a harmonious bite and facial profile. This approach is particularly advantageous in complex cases where precise control of the maxillary position is necessary.

Conversely, the mandible first approach starts with the repositioning of the mandible. This technique is sometimes preferred when the primary deformity is mandibular or when there is a significant discrepancy in the mandibular position. By first addressing the mandible, surgeons can ensure that the mandible’s new position provides a stable and functional foundation for subsequent maxillary adjustments. This approach can be beneficial in cases where mandibular movements are less complex or when the mandibular correction is expected to significantly influence the maxillary position [1].

## Innovative approach with virtual surgical planning

Orthognathic surgery has undergone enormous change in recent decades. As part of the increasing digital transformation of medicine, the planning and execution of orthognathic surgery is changing significantly due to developments in the field of virtual surgical planning (VSP) and 3D printing. Instead of classic two-dimensional imaging, three-dimensional imaging is carried out using CT or cone beam computed tomography (CBCT). This DICOM data is then converted into an STL file, which is used to create a digital model. Using this digital model, the planned movements of the jaws can be carried out virtually in special programs. In combination with current capabilities in 3D printing, the three-dimensional models can be printed to provide visualization for the patient, and splints can be manufactured. Using these splints, the virtually planned operation can be accurately replicated on the patient during surgery. These techniques offer enhanced accuracy compared to traditional methods. Barone et al. conducted a comparison between postoperative outcomes and planned jaw movements using established osseous reference benchmarks. They found that surgeries planned with VSP showed a significantly lower deviation from the intended values than the operations planned with TSP [8]. Eliminating the need for an external laboratory for planning and model production, coupled with improved interdisciplinary collaboration through the digital approach, reduces planning time. This decreases reliance on other specialized departments, and research indicates that VSP proves to be a more efficient alternative, despite the initial investment in high-performance computers and software. However, it is important to critically assess the necessity for surgeon training, as they are responsible for the entire digital planning process [3, 6, 7, 9].

## Patient-specific implants and guides

Patient-specific implants (PSIs) and guides also represent significant advancements in orthognathic surgery, offering more precision and efficiency. These technologies have been implemented in routine practice for several years, significantly improving surgical outcomes.

PSIs are designed and manufactured based on the patient’s specific anatomical data, allowing for individualized treatment. The use of these implants eliminates the need for intraoperative adjustments, leading to reduces surgery time and enhanced accuracy. Similarly, patient-specific guides, created using preoperative data, facilitate precise osteotomies and jaw repositioning. These guides help replicate the virtual surgical plan accurately in the operating room, minimizing the risk of errors and improving postoperative results 10], [11], [12.

## Perspectives in orthognathic surgery

The ongoing introduction of new technologies enables constant progress in orthognathic surgery in terms of interdisciplinary collaboration, optimization of the planning process and time management. Enhancing the quality of treatment outcomes in the future is equally significant. Current relevant developments include artificial intelligence (AI), patient-specific implants (PSI), mixed reality (MR) and deep learning approaches.

The use of AI in orthognathic surgery is increasingly investigated and seen as a promising technology. AI can help to identify anatomical structures, make differential diagnoses or predict blood loss and changes in facial morphology and soft tissues. Especially VSP can be significantly shortened as a result, which considerably optimizes the cost-effectiveness of this method [13, 14].

As even 3D-printed splints are a potential cause of inaccuracies, research is being carried out into concepts for splintless positioning and fixation. PSI, which are specially designed and manufactured for each patient, represent a more precise alternative. This enables individual positioning of the osteotomized jaw segments, eliminating the need for splints and offering an accurate translation from VSP to the operating room. The PSI are planned virtually preoperatively using specialized software programs. The implants are then manufactured using 3D printing. These PSIs are proven to be beneficial, especially in severe transverse deficits or asymmetric cases. Further developments of PSI designs could particularly improve the precision of segmental maxillary osteotomies [11, 12].

Mixed reality (MR) also enables splintless positioning, allowing the physical and digital worlds to merge seamlessly by projecting virtual objects into the user’s field of vision. As MR is compatible with virtual surgical planning data sets, surgeons can already benefit from MR during pre-operative planning and better understand complex interventions. This is achieved by providing real-time patient data, replacing physical cutting guides with direct projection of the guide lines onto the anatomy and providing interactive feedback on the progress of the procedure [15, 16].

Despite the significant potential offered by the aforementioned technological advancements, there remain numerous obstacles and constraints that complicate their application in orthognathic surgery. Key concerns include economic viability, duration of preparation and planning, and technological limitations. The expertise of software developers and medical professionals, along with their technical proficiency, will remain essential for establishing an affordable, feasible, and uniform integration of these technologies into routine medical practice. One potential solution could involve implementing a completely automated digital workflow utilizing deep learning, thereby streamlining processes and facilitating usage, even for individuals with limited expertise [16, 17].

## Summary

Orthognathic surgery, a branch of oral and maxillofacial surgery, corrects acquired and congenital anomalies in the shape and position of the jaws, associated with functional and aesthetic impairments. Traditional treatment approaches involve a combination of orthodontic therapy and surgical interventions, which can be time-consuming and prone to error. With the introduction of digital technologies such as VSP and 3D printing, clinical outcomes can be improved through increased accuracy and efficiency of treatment, allowing for more precise planning and reducing dependence on external laboratories. Future developments, including the use of AI and PSI, hold promise for further advancements in the treatment of dysgnathia. Despite the potential of these technologies, challenges such as time and economic efficiency and technical limitations remain.
